# The Neural Signature of Visual Learning Under Restrictive Virtual-Reality Conditions

**DOI:** 10.3389/fnbeh.2022.846076

**Published:** 2022-02-16

**Authors:** Gregory Lafon, Haiyang Geng, Aurore Avarguès-Weber, Alexis Buatois, Isabelle Massou, Martin Giurfa

**Affiliations:** ^1^Research Center on Animal Cognition, Center for Integrative Biology, CNRS, University of Toulouse, Toulouse, France; ^2^College of Animal Sciences (College of Bee Science), Fujian Agriculture and Forestry University, Fuzhou, China; ^3^Institut Universitaire de France, Paris, France

**Keywords:** vision, visual learning, virtual reality, honey bee (*Apis mellifera*), brain, IEG expression, mushroom bodies, optic lobes

## Abstract

Honey bees are reputed for their remarkable visual learning and navigation capabilities. These capacities can be studied in virtual reality (VR) environments, which allow studying performances of tethered animals in stationary flight or walk under full control of the sensory environment. Here, we used a 2D VR setup in which a tethered bee walking stationary under restrictive closed-loop conditions learned to discriminate vertical rectangles differing in color and reinforcing outcome. Closed-loop conditions restricted stimulus control to lateral displacements. Consistently with prior VR analyses, bees learned to discriminate the trained stimuli. *Ex vivo* analyses on the brains of learners and non-learners showed that successful learning led to a downregulation of three immediate early genes in the main regions of the visual circuit, the optic lobes (OLs) and the calyces of the mushroom bodies (MBs). While *Egr1* was downregulated in the OLs, *Hr38* and *kakusei* were coincidently downregulated in the calyces of the MBs. Our work thus reveals that color discrimination learning induced a neural signature distributed along the sequential pathway of color processing that is consistent with an inhibitory trace. This trace may relate to the motor patterns required to solve the discrimination task, which are different from those underlying pathfinding in 3D VR scenarios allowing for navigation and exploratory learning and which lead to IEG upregulation.

## Introduction

Learning relies on changes in neural activity and/or connectivity in the nervous system, which underlie the acquisition of new, durable information based on individual experience. Invertebrate models have proved to be extremely influential to characterize learning and memory at multiple levels, not only because they allow determining where and when such changes occur in the nervous system (Kandel, 2001; Heisenberg, 2003; Giurfa, 2007, 2013, 2015; Benjamin et al., 2008; Cognigni et al., 2018) but also because their behavioral performances can be studied in standardized laboratory protocols that allow full control over the sensory variables that animals should learn and memorize. A paradigmatic example is provided by the honey bee *Apis mellifera*, where the study of olfactory learning and memory experienced significant progresses thanks to the availability of a Pavlovian conditioning protocol that offers the possibility of acquiring consistent behavioral data coupled with the simultaneous use of invasive methods to record neural activity (Menzel, 1999, 2012; Giurfa, 2007; Giurfa and Sandoz, 2012). In this protocol, termed the olfactory conditioning of the proboscis extension reflex (PER), harnessed bees learn to associate an odorant with a reward of sucrose solution (Bitterman et al., 1983; Giurfa and Sandoz, 2012). The immobility imposed to the trained bees and the Pavlovian nature of the association learned (the odorant acts as the conditioned stimulus and the sucrose reward as the unconditioned stimulus) allows a full control over the stimulations provided and thus a fine characterization of behavioral changes due to learning and memory.

In the case of visual learning by honey bees, this possibility is reduced as performances are mostly studied in free-flying foragers (Giurfa, 2007; Avargues-Weber et al., 2011) under semi-natural conditions. Yet, virtual-reality (VR) environments have been recently developed to overcome this limitation (Schultheiss et al., 2017) as they provide not only a full control over the visual surrounding of an experimental subject, be it tethered or not, but also the delivery of physically impossible ambiguous stimuli, which give conflicting visual information (Frasnelli et al., 2018). In one type of VR that we developed in the last years, a tethered bee walks stationary on a treadmill while being exposed to a controlled visual environment displayed by a video projector. Bees can then be trained with virtual targets that are paired with gustatory reward or punishment (Buatois et al., 2017, 2018; Rusch et al., 2017, 2021; Schultheiss et al., 2017; Zwaka et al., 2018). To create an immersive environment, closed-loop visual conditions are used in which the variations of the visual panorama are determined by the walking movements of the bee on the treadmill. Under these conditions, bees learn and memorize simple (Buatois et al., 2017, 2018) and higher-order (Buatois et al., 2020) visual discriminations, which offers the potential for mechanistic analyses of visually oriented performances (Zwaka et al., 2018; Rusch et al., 2021).

We have used two different types of closed loop situation so far: a restrictive 2D situation, in which bees can displace conditioned targets only frontally (i.e., from left to right and vice versa) (Buatois et al., 2017, 2018, 2020), and a more realistic 3D situation which includes a depth dimension so that targets expand upon approach and retract upon distancing (Lafon et al., 2021). Although bees learn to discriminate color stimuli in both conditions, the processes underlying such learning may differ given the different conditions imposed to the bees in terms of stimulus control. Indeed, while in 3D scenarios movement translates into a displacement and a recognizable change in the visual scene, which can then be computed against the available internal information about the displacement, 2D scenarios are restricted to the execution of actions that are dependent on reinforcement contingency. These two conditions may give rise to different mechanisms of information acquisition.

In a recent work, we studied color learning in the 3D scenario and quantified immediate early genes (IEGs) in the brain of learners and non-learners to uncover the regions that are involved in this discrimination learning (Geng et al., 2022). IEGs are efficient markers of neural activity as they are transcribed transiently and rapidly in response to specific stimulations inducing neural activity without *de novo* protein synthesis (Clayton, 2000; Minatohara et al., 2015; Bahrami and Drabløs, 2016). Three IEGs were quantified on the basis of numerous reports that associated them with foraging and orientation activities (Kiya and Kubo, 2011; Shah et al., 2018; Singh et al., 2018; Ugajin et al., 2018; Iino et al., 2020): *kakusei*, a nuclear non-coding RNA (Kiya et al., 2007), the hormone receptor 38 gene (*Hr38*), a component of the ecdysteroid signaling pathway (Fujita et al., 2013), and the early growth response gene-1 (*Egr1*), which is a major mediator and regulator of synaptic plasticity and neuronal activity (Duclot and Kabbaj, 2017). We found that color learning in the 3D VR environment was associated with an *upregulation* of *Egr1* in the calyces of the mushroom bodies (Geng et al., 2022), a main structure of the insect brain repeatedly associated with the storage and retrieval of olfactory memories (Heisenberg, 2003; Menzel, 2012). No other changes of IEG expression were detected in other regions of the brain, thus underlining the relevance of mushroom bodies for color learning and retention (Geng et al., 2022).

Here we asked if color learning in the more restrictive 2D VR induces changes in IEG expression, both at the gene level and at the brain region level, similar to those detected in the 3D VR system. Asking this question is important to determine if changes in IEG expression differ according to the degrees of freedom of the VR system and the distinct motor patterns that are engaged in either case. Despite the similarity in behavioral performance (bees learn to discriminate colors in both scenarios), we reasoned that the processes underlying learning may be different given the restrictive conditions of the 2D VR, which demand a tight stimulus control while the 3D VR enables exploration of the virtual environment. We thus studied color learning in the 2D VR environment and performed *ex vivo* analyses to map IEG expression in brain areas of learners and non-learners, which had the same sensory experience and only differed in terms of learning success.

## Results

### Behavioral Analyses

**FIGURE 1:**
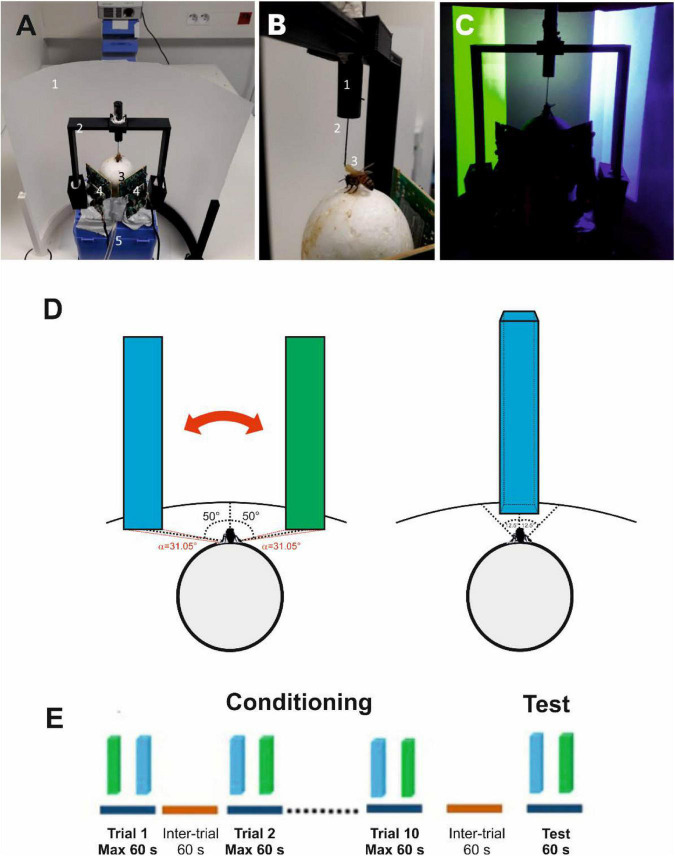
Experimental setup, choice criterion and conditioning procedure. **(A)** Global view of the setup. 1, semicircular projection screen made of tracing paper; 2, holding frame to place the tethered bee on the treadmill; 3, the treadmill was a Styrofoam ball positioned within a cylindrical support (not visible) floating on an air cushion; 4, infrared mouse optic sensors allowing to record the displacement of the ball and to reconstruct the bee’s trajectory; 5, air arrival. The video projector displaying images on the screen from behind can be seen on top of the image. **(B)** The tethering system. 1, plastic cylinder held by the holding frame; the cylinder contained a glass cannula into which a steel needle was inserted; 2, the needle was attached to the thorax of the bee; 3, its curved end was fixed to the thorax by means of melted bee wax. **(C)** Color discrimination learning in the VR setup. The bee had to learn to discriminate two vertical bars based on their different color and their association with reward and punishment. Bars were green and blue on a dark background. Color intensities were adjusted to avoid phototactic biases independent of learning. Displacement of the bars was restricted to the 2D plane in front of the bee. **(D)** Left: view of the stimuli at the start of a trial or test. The green and the blue virtual bars were a presented at –50° and + 50° of the bee’s longitudinal axis of the bee. Stimuli could be only displaced by the bee from left to right and vice versa (double red arrow). The red angles on the virtual surface indicate the visual angle subtended by each bar at the bee position (α = 31.05°). Right: Choice of a bar. A choice was recorded when the bee kept the center of the object between –12.5° and + 12.5° in front of it for 1 s. The bar image was then frozen during 8 s and the corresponding reinforcement (US) was delivered. **(E)** Conditioning protocol. Bees were trained along 10 conditioning trials that lasted a maximum of 1 min and that were spaced by 1 min (intertrial interval). After the end of conditioning, and following an additional interval of 1 min, bees were tested in extinction conditions during 1 min.

Honey bee foragers from a hive located in our apiary were captured at an artificial feeder to which they were previously trained. They were enclosed in individual glass vials and brought to the laboratory where they were prepared for the VR experiments. A tether was glued on their thorax (Figures 1A,B), which allowed to attach them to a holder to adjust their position on a treadmill. The treadmill was a polystyrene ball that was suspended on an air cushion produced by an air pumping system (see section “Materials and Methods” for details). The bee suspended from its tether could walk stationary on the treadmill; its movements were recorded by two infrared optic-mouse sensors placed on the ball support perpendicular to each other, which allowed to reconstruct the trajectories and quantify motor parameters. A semi-cylindrical screen made of semitransparent tracing paper was placed in front of the treadmill (i.e., of the walking bee; Figure 1A). Images were projected onto this screen by a video projector placed behind it.

Bees were trained to discriminate a green from a blue vertical bar against a black background during ten conditioning trials (Figure 1C; see Supplementary Figure 1 for color characteristics). Experiments were performed under 2D closed-loop conditions so that the movements of the walking bee displaced the bars laterally on the screen to bring them toward or away from front of the bee. During training, one of the bars (CS+) was rewarded with 1 M sucrose solution while the other bar (CS−) was punished with an aversive 3M NaCl solution (Ayestaran et al., 2010; de Brito Sanchez et al., 2015; Aguiar et al., 2018). A choice was recorded when the bee moved one rectangle to the center of the screen (i.e., between −12.5° and +12.5° of the bee’s central axis; see Figure 1D, right).

We segregated learners and non-learners according to the bees’ performance in a dedicated unrewarded test at the end of the training. Learners (*n* = 23) were those bees that showed successful discrimination in the test (i.e., which chose the CS +). Non-learners (*n* = 17), were those bees that did not succeed in the test (i.e., they either chose the CS− or did not make a choice). Importantly, these bees have the same sensory experience in terms of exposure to the color stimuli and reinforcements as our training procedure froze the CS + or the CS− stimuli in front of the bee during 8 s upon a choice and delivered the reinforcements accordingly. Bees that did neither choose the CS + nor the CS− in at least five trials were excluded from the analysis.

**FIGURE 2:**
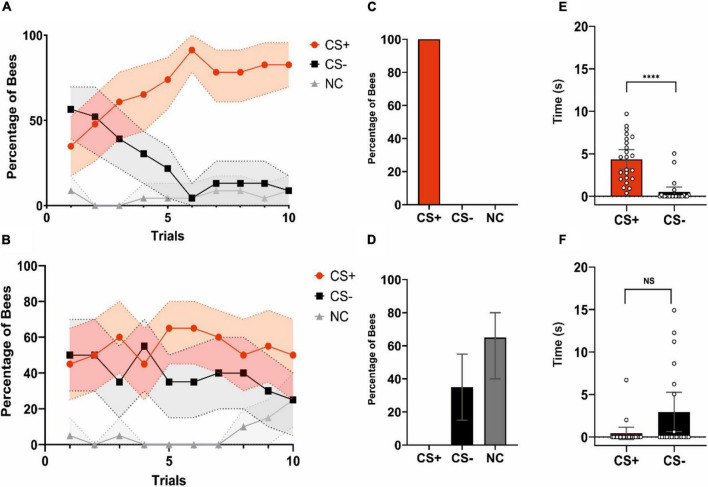
Acquisition and test performances of learners and non-learners. **(A)** Acquisition performance of learners (i.e., bees that chose the CS + in the non-reinforced test; *n* = 23). The red, black and gray curves show the percentages of bees choosing the CS +, CS–, or not making a choice (NC), respectively. Bees learned the discrimination between CS + and CS–. **(B)** Acquisition performance of non-learners (i.e., bees that chose the CS– or did not make a choice in the non-reinforced test; *n* = 17). These bees did not learn to discriminate the CS + from the CS–. **(A,B)** Shaded areas around curves indicate the 95% confidence interval. **(C)** Test performance of learners (% of bees choosing either the CS +, the CS– or not making a choice). Per definition these bees only chose the CS + first. **(D)** Test performance of non-learners. (% of bees choosing either the CS+, the CS– or not making a choice). Per definition these bees chose either the CS– or did not make a choice (NC). **(C,D)** Error bars represent the 95% confidence interval. **(E)** Time (s) spent by learners fixating the CS + and the CS– during the test. Learners spent more time fixating the CS + consistently with their stimulus choice. Bars represent the time spent keeping the object within –12.5°/ + 12.5° in front of the bee. Scatter plots represent individual fixation times, ****p < 0.0001. **(F)** Time (s) spent by non-learners fixating the CS + and the CS– during the test. Non-learners did not differ in their fixation time of the CS + and the CS–. Bars represent the time spent keeping the object within –12.5°/ + 12.5° in front of the bee. Scatter plots represent individual fixation times. NS, non-significant. **(E,F)** Error bars represent the 95% confidence interval.

Acquisition was significant for learners during conditioning trials (Figure 2A; CS*Trial effect: χ2 = 47.746, df:2, *p* < 0.0001), thus showing that the categorization made based on test performance reflected well learning success. The percentages of bees responding to the CS + and to the CS− differed significantly along trials (CS + vs. CS−: CS*Trial; *z* = 6.845, *p* < 0.0001). Significant differences were also found between the bees responding to the CS− and the non-responders (CS− vs. NC: CS*Trial; *z* = 3.541, *p* = 0.0004) but not between bees responding to the CS + and non-responders (CS + vs. NC: CS*Trial; *z* = −1.201, *p* = 0.23). Non-learners (*n* = 17) did also show a significant CS*Trial effect (Figure 2B; χ2 = 9.8383, df:2, *p* = 0.007), but this effect was introduced by the non-responders. These bees differed significantly along trials both from the bees responding to the CS + (CS + vs. NC: CS*Trial; *z* = 2.356, *p* = 0.019) and from the bees responding to the CS− (CS− vs. NC: CS*Trial; *z* = 3.068, *p* = 0.002). On the contrary, the percentages of bees responding to the CS + and to the CS− did not vary along trials (CS + vs. CS−: CS*Trial; *z* = 1.437, *p* = 0.2), consistently with the absence of learning.

Learners and non-learners did not differ in their motor activity during training, thus excluding this factor as determinant of possible changes in neural activity. When walking speeds and the distances traveled were compared between groups, no significant differences were detected (*Distance*: Group; χ2 = 1.93, df:1, *p* = 0.16; *Speed*: Group; χ2 = 1.78, df:1, *p* = 0.18).

In the non-reinforced test, per definition learners (Figure 2C) chose correctly the CS + (100% of the bees) while non-learners (Figure 2D) did either chose the CS− (35%) or did not perform any choice (65%). Learners spent more time fixating the CS + than the CS− consistently with the choice made during the test (Figure 2E; Wilcoxon signed rank exact test: V = 17, *p* < 0.0001) while non-learners did not differ in their fixation time for both stimuli in spite of a tendency to fixate more the CS− (Figure 2F; V = 26, *p* = 0.05).

### Molecular Analyses

**FIGURE 3:**
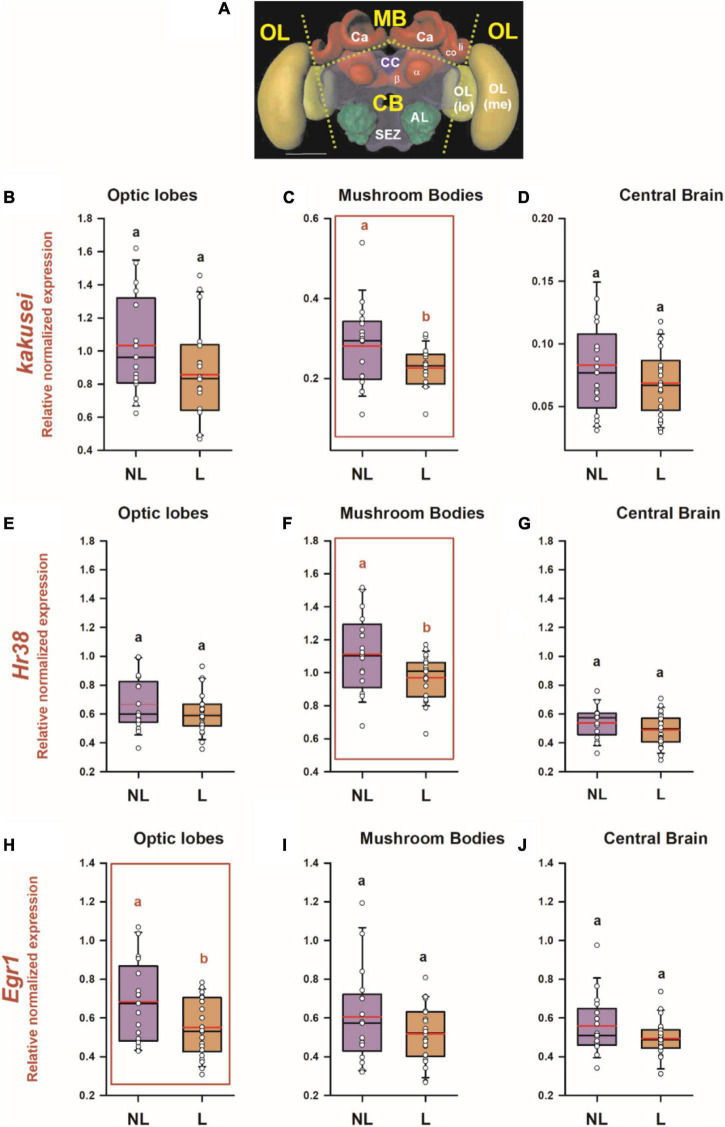
Differential IEG expression as a consequence of associative color learning in a 2D VR environment. **(A)** Honey bee brain with sections used for quantifying IEG expression. Yellow labels indicate the brain regions used for the analysis: MB, mushroom body; CB, central brain; OL, optic lobes. The dashed lines indicate the sections performed. Ca, calyx of the mushroom body; li, lip; co, collar; a and b, a and b lobes of the mushroom body; CC, central complex; AL, antennal lobe; SEZ, suboesophagic zone; OL, optic lobe; Me, medulla; lo, lobula. Relative normalized expression of panels **(B–D)** *kakusei*, **(E–G)** *Hr38*, and **(H–J)** *Egr1* in three main regions of the bee brain, optic lobes **(B,E,H)**, calyces of the mushroom bodies **(C,F,I)**, and central brain **(D,G,J)**. The expression of each IEG was normalized to the geometric mean of *Actin and Ef1a* (reference genes). IEG expression was analyzed in individual brains of bees belonging to two categories: *learners* (L: conditioned bees that responded correctly and chose the CS+ in their first choice during the non-reinforced test) and *non-learners* (NL: conditioned bees that did not choose the CS + in their first choice during the non-reinforced test). The range of ordinates was varied between panels to facilitate appreciation of data scatter. In all panels, *n* = 22 for learners and *n* = 17 for non-learners. Different letters on top of box plots indicate significate differences (two-sample *t* test; *p* < 0.05). Box plots show the mean value in red. Error bars define the 10th and 90th percentiles. Red boxes indicate cases in which significant variations were detected.

We aimed at determining if visual learning in the 2D VR induces transcriptional changes revealing the neural trace of the associative learning described in the previous section. To this end, we performed RT-qPCR in individual brains of learners (*n* = 22; one learner brain was lost during the dissection process) and non-learners (*n* = 17), focusing on three main brain sections (Figure 3A): the optic lobes (OLs), the calyces of the mushroom bodies (MB) and the remaining central brain (CB), which included mainly the central complex, the subesophageal zone and the peduncula of the mushroom-bodies (a and b lobes). Brains were collected 1 h after the retention test, which ensures that expression of all three genes was already induced (typically, from 15 to 90 min in the case of kakusei (Kiya et al., 2007; Ugajin et al., 2012) and 30–60 min in the case of *Hr38* and *Egr1* (Ugajin et al., 2018; Iino et al., 2020)).

Two reference genes were used for the normalization (see Table 1): *Ef1a* (E = 106%) and *Actin* (E = 110%) (Marchal et al., 2019). Within-brain structure analyses showed that reference genes did not vary between learners and non-learners (*t* test; all comparisons NS; see Supplementary Figure 3) thus enabling further comparisons between these two categories with respect to the three target IEGs. To this end, the normalization procedure used the geometric mean of the two reference genes. No cross-comparisons between brain regions or genes were performed.

**TABLE 1:** Primer sequences used to quantify RNA expression of genes of interest and reference genes by RT-qPCR.

Type of gene	Target	Primer sequence 5’ 3’	Amplicon length (bp)	E (%)	*R* ^2^
Target genes	*Kakusei*	CTACAACGTCCTCTTCGATT (forward) CCTACCTTGGTATTGCAGTT (reverse)	149	96.4	0.991
	*Hr38*	TGAGATCACCTGGTTGAAAG (forward) CGTAGCAGGATCAATTTCCA (reverse)	118	106	0.995
	*Egr1*	GAGAAACCGTTCTGCTGTGA (forward) GCTCTGAGGGTGATTTCTCG (reverse)	138	109	0.991
Reference genes	*Ef1a*	AAGAGCATCAAGAGCGGAGA (forward) CACTC TTAATGACGCCCACA (reverse)	148	106	0.993
	*Actin*	TGCCAACACTGTCCTTTCTG (forward) AGAATTGACCCACCAATCCA (reverse)	156	110	0.995

*bp, amplicon length; E (%), efficiency; R^2^, coefficient of correlation obtained for the standard curve; Hr38, hormone receptor 38 gene; Egr1, early growth response gene-1; Ef1α, elongation factor 1 α gene.*

Figures 3B–J show the relative normalized expression of *kakusei, Hr38* and *Egr1*, respectively, for the three brain regions considered in the case of *learners and non-learners.* Significant variations of normalized expression between learners and non-learners were found for the three IEGs: in the case of *kakusei* and *Hr38*, these differences were restricted to the MBs (*kakusei:* Figure 3C; two-sample *t* test; *t* = −2.23; df:37; *p* = 0.03; *Hr38:* Figure 3F; *t* = −2.39; df:37; *p* = 0.02) while in the case of *Egr1*, they were observed in the optic lobes (*Egr1:* Figure 3H; *t* = −2.32; df:37; *p* = 0.03). All other within-structure comparisons between learners and non-learners were not significant (*p* > 0.05). Notably, in the three cases in which significant variations of IEG expression were found, learners exhibited a *downregulation* of IEG expression with respect of non-learners. In addition, from the three cases, two referred to the MB calyces, which indicates the important role of this region for visual learning and memory.

## Discussion

The present work studied visual learning under a restrictive 2D VR environment and confirmed that bees can learn to discriminate visual stimuli based on their color under these artificial conditions. Walking parameters did not differ between learners and non-learners so that changes in IEG expression could be ascribed to learning success. We showed that associative color learning leads to a downregulation of the three IEGs considered in different areas of the visual circuit. While *Egr1* was downregulated in the optic lobes, *Hr38* and *kakusei* were coincidently downregulated in the MB calyces. Our work thus reveals that the neural trace of associative color learning in the bee brain is distributed along the sequential pathway of color processing and highlights the importance of MBs for color learning in bees.

### Immediate Early Genes Downregulation in the Bee Brain

We observed an IEG *downregulation* both in the optic lobes and the calyces of the MBs. This phenomenon is interesting as increased neural activity resulting from experience-dependent phenomena is usually reflected by an *upregulation* of IEG expression (Bahrami and Drabløs, 2016). Typically, upon neural responses, a relatively rapid and transient pulse of gene expression may occur, which corresponds to an experience-dependent activation of the underlying synaptic circuitry (Clayton, 2000; Okuno, 2011). In our case, however, the downregulation observed indicates that a different form of experience-dependent change in neural activity occurred as a consequence of learning. A possible explanation for this phenomenon may put the accent on an inhibition of neural activity in key visual areas – optic lobes and mushroom bodies - of the learner group.

In the optic lobes, *Erg1* downregulation may correspond to an increased GABAergic inhibitory activity associated with learning. The optic lobes exhibit multiple GABAergic fibers distributed principally in the medulla and the lobula (Schäfer and Bicker, 1986) so that neural activity in these regions is subjected to intense GABAergic inhibitory signaling. Higher GABAergic activity has been reported in the optic lobes of forager bees *via* quantification of *Amgad*, the honey bee homolog of the gene responsible for synthesizing the enzyme GAD (Kiya and Kubo, 2010), which catalyzes the decarboxylation of glutamate to GABA. This increase was accompanied by an increase in *kakusei* (Kiya and Kubo, 2010), which we did not observe. Yet, we did not study foraging behavior in a natural context, but associative learning in a controlled laboratory context. Natural foraging may involve multiple behavioral components and stimulations that may be responsible for the increase of *kakusei* that was absent in our study. The interesting finding is, however, that *Amgad* expression revealed higher GABAergic neuron activity in the optic lobes of foragers, confirming the importance of inhibitory feedback for sustaining experience-dependent visual responses. This conclusion is supported by observed increases of GABA titers in the honey bee optic lobes upon restart of foraging activities (Chatterjee et al., 2021) and by findings in fruit flies indicating that GABA-ergic neurons in the optic lobes are involved in tuning the sensitivity and selectivity of different visual channels (Keles and Frye, 2017; Keles et al., 2020).

In the calyces of the MBs, where coincident downregulation of *kakusei* and *Hr38* was found, neural inhibition is provided by GABAergic feedback neurons (the so-called Av3 neurons) (Rybak and Menzel, 1993), which are responsible for the sparse coding responses exhibited by Kenyon cells, the constitutive neurons of the MBs. Similar GABAergic neurons exist in fruit flies, which provide inhibitory feedback to the MBs. These neurons, termed APL (anterior paired lateral) neurons, are necessary for discrimination learning of similar odorants. When flies are trained to discriminate odorants in a simple differential conditioning, disrupting the Kenyon cell-APL feedback loop decreases the sparseness of Kenyon cell odor responses, increases inter-odor correlations and prevents flies from learning to discriminate similar, but not dissimilar, odors (Lin et al., 2014). Inhibitory feedback onto the calyces of honey bees is needed for solving patterning tasks in which insects have to suppress summation of responses to single elements previously rewarded when they are presented in an unrewarded compound (Devaud et al., 2015) (i.e., animals have to learn to respond to the elements and not to the compound) or for reversal learning (Boitard et al., 2015). A similar conclusion applies to fruit flies as GABAergic input to the MBs provided by APL neurons also mediates the capacity to solve patterning tasks (Durrieu et al., 2020). Increased feedback inhibition at the level of the MBs may therefore appear as a hallmark of certain learning phenomena, which require enhanced neural sparseness to decorrelate stimulus representations and thus memory specificity. In our experiments, both *kakusei* and *Hr38* were subjected to downregulation in the MBs as a consequence of learning, a phenomenon that may be due to plastic changes in GABAergic signaling in the calyces of the MBs. Importantly, other visual areas such as the central complex (Honkanen et al., 2019) or the anterior optic tuberculum (Mota et al., 2011, 2013), among others, could exhibit similar variations undetectable for our methods as sectioning the frozen bee brain for molecular analyses does not allow a fine dissection of these areas.

Immediate early genes downregulation is not a common phenomenon as upregulation is usually reported to indicate the presence of neural activation (Geng et al., 2022). Our hypothesis on neural inhibition being the cause for this downregulation requires, therefore, to be considered with caution. Further experiments are necessary to validate it, using – for instance – electrophysiological recordings in key areas of the visual circuits of learners to verify that neural activity is indeed sparser therein compared to non-learners. In addition, quantifying IEG expression in preparations in which neural inhibition has been characterized extensively at the cellular level such as in the case of hippocampal and cerebellum slices exhibiting long-term depression (LTD) (Massey and Bashir, 2007) could be also important.

### The Neural Signature of Associative Learning Differs Between Different Forms of Virtual Reality

While the main finding in our experiments refer to a downregulation of IEG genes in key regions of the visual circuit, our previous work using a different 3D VR system yielded a different result (Geng et al., 2022). In this 3D VR, bees could explore the virtual surroundings around the stimuli to be learned (not bars, but cuboids that expanded upon forward movements of the bee) and could displace these stimuli laterally and in depth. They explored and learned to discriminate the color stimuli proposed to them and their learning success was comparable, yet slightly lower than that observed in the 2D VR arena (50% vs. 55%, respectively). IEG analyses comparing learners and non-learners in the 3D VR reported an *upregulation* of *Egr1* expression in the MB calyces of learners but not of non-learners. No other change was detected for *kakusei* and *Hr38* in the same three brain regions considered in the present work (Geng et al., 2022).

These differences are difficult to interpret as the 2D and the 3D VR experiments were not done simultaneously but in different years, though in similar seasons. In both cases, motivated foragers captured at a feeder were used for the experiments. The previous visual experience of these foragers may have differed across individual and between years, thus leading to differences in performances. This explanation seems, however, rather implausible given that in bees rely on the most recent appetitive learning as the one guiding predominantly actual choices. In addition, irrespective of differences in the VR environments and the resulting difference in VR immersivity, the experiments were done under similar handling conditions and using strictly the same behavioral criteria. Gene analyses were also performed under the same conditions and using the same materials and methods. Thus, the contrasting results obtained in the two VR scenarios may be due to the distinct constraints they imposed to achieve discrimination learning and to the fact that the two scenarios may engage different acquisition mechanisms for learning visual information. In the 3D scenario, bees explored both the stimuli – the vertical color cuboids – and the imaginary empty surroundings; they could return to the stimuli if they missed them and walk around them, which added an important exploratory component that was absent in the 2D arena. In the latter case, although bees could also bring back the stimuli if they missed them by walking too fast, such a control was restricted to the frontal plane and did not allow for three-dimensional inspection. *Erg1* upregulation in the 3D VR upon learning may thus reflect the convergent effects of an exploratory drive and learning in a non-constrained environment. It cannot be due to a pure exploration of the environment as non-learners exhibited the same motor performances and did not show *Egr1* upregulation.

In the 2D VR, bees were forced to control tightly the lateral displacements of the stimuli – the color rectangles – without any further change allowed. This environment and task may thus impose a higher stimulus and movement control and force the bee to focus exclusively and artificially on lateral stimulus movements to gain access to sucrose reward and avoid aversive saline solution. Although in both VR scenarios the background was empty and only the training stimuli were visible, the 2D VR missed the expansion of the images upon approach and thus lacked of immersivity. In this context, GABA-mediated inhibition may act as a gain control mechanism that enhances response efficiency and stimulus control. In the primary visual cortex (V1) of vertebrates, GABA inhibition has been proposed to play a fundamental role in establishing selectivity for stimulus orientation and direction of motion (Rose and Blakemore, 1974; Sillito, 1979; Tsumoto et al., 1979). As the latter is particularly important in the 2D VR, enhanced GABA inhibition could be associated with learning to master the visual discrimination in this context.

In addition, a different, yet compatible explanation for the different patterns of IEG expression found in the 3D and the 2D VR refers to a possible difference in the visual acquisition mechanisms recruited by these two scenarios. In a navigation task, body movement translates into a displacement and a recognizable change in the visual scene, which can then be computed against the available internal information about the displacement (von Holst and Mittelstaedt, 1950). These pathfinding, closed-loop actions can be viewed as different from motor actions that are contingent on reinforcement such as operant behaviors produced when a visual discriminative stimulus is present (Skinner, 1938). In the latter case, vision is also engaged in discrimination learning but in a context that is not navigational. Visual learning in the 2D VR could be seen as a form of operant learning in which colors define the action to be produced to obtain the appropriate reinforcement. Thus, the observed difference in IEG expression between the two types of VR may reflect a difference in the mechanisms used to reach the rewarded stimulus.

### The Role of Mushroom Bodies for Visual Learning and Memory

Our work highlights the participation of mushroom bodies in visual learning and short-term visual retention. Numerous works have demonstrated the necessity of these brain structures for the acquisition, storage and retrieval of olfactory memories in bees (Menzel, 1999, 2014; Devaud et al., 2015) and other insects (Heisenberg, 2003; Guven-Ozkan and Davis, 2014; Cognigni et al., 2018). Yet, less is known about their implication in visually driven behavioral and neural plasticity (Avarguès-Weber et al., 2012; Avargues-Weber and Mota, 2016). In our study, the full control over sensory stimulation offered by the VR system allowed a sound comparison between the brain of learners and non-learners, which revealed a neural signature for visual learning that included the mushroom bodies.

The implication of mushroom bodies in visual learning and memory in the bee is expected given the parallels between visual and olfactory inputs at the level of the calyces. While afferent projection neurons convey olfactory information to the lip, a subdivision of the calyces (Kirschner et al., 2006), afferent neurons from the lobula and the medulla, which are part of the optic lobes, convey visual information to other calyx subdivisions, the collar and the basal ring (Ehmer and Gronenberg, 2002; Paulk et al., 2009). In spite of this similarity, studies addressing the role of mushroom bodies in honey bee visual learning and memory remain rare.

Bees learning to associate color lights with the presence or absence of an electric shock in a double-compartment box (Kirkerud et al., 2017; Marchal et al., 2019) require the ventral lobe of the mushroom bodies to learn to avoid the punished color and spend more time in the safe color (Plath et al., 2017). In the same study, pharmacological blockade of one of the four collars (two per MB) had no effect on discrimination learning (Plath et al., 2017), which does not exclude a participation of this MB region in this visual learning given that the remaining three calyces could compensate for the loss of the blocked one. In a different study, upregulation of the dopamine receptor *Amdop1* was found in the calyces of the MBs when bees were trained to inhibit positive phototaxis toward a colored light (Marchal et al., 2019).

More recently, the implication of MBs in visual navigation was shown in wood ants *Formica rufa*, which are innately attracted to large visual cues (i.e., a large vertical black rectangle) and which can nevertheless be trained to locate and travel to a food source placed at a specific angle away from the attractive black rectangle (Buehlmann et al., 2020). When their MB calyces were blocked by injection of procaine (Muller et al., 2003; Devaud et al., 2007), ants reverted their trajectories toward the attractive rectangle, which suggests a role for mushroom bodies in the dissociation between innate and learned visual responses, and in navigational memory (Buehlmann et al., 2020). In another study involving the ant *Myrmecia midas*, procaine was again used to block MB function *via* delivery into the vertical lobes and evaluate the impact of this blockade in orientation in a familiar environment (Kamhi et al., 2020). Experienced forager with procaine-inactivated VLs had tortuous paths and were unable to find their nest, whereas control ants were well directed and successful at returning home (Kamhi et al., 2020). Overall, these two studies on ant navigation indicate that the vertical lobes of MBs are necessary for retrieving visual memories for successful view-based navigation.

Studies on the role of MBs for visual learning and memory in fruit flies have yielded contradictory findings. Mushroom body deficits do not affect learning success in the flight simulator, a setup in which tethered flies in stationary flight learn to avoid quadrants associated with specific visual landmarks based on the presence of an aversive heat beam pointed toward their thorax (Wolf et al., 1998). Similarly, learning to discriminate colors in a cylindrical container made of a blue-lit and a yellow-lit compartment, one of which was associated with aversive shaking of the flies, was not affected in mushroom body mutants (Heisenberg et al., 1985). Spatial learning of a non-heated spot in an otherwise heated cylindrical arena displaying surrounding visual landmarks is possible in the absence of functional mushroom bodies but not of the central complex (Ofstad et al., 2011). Although these various results points toward a dispensability of MBs for visual learning in fruit flies (Wolf et al., 1998), experiments comparing appetitive and aversive color learning and discrimination question this view (Vogt et al., 2014). When blue and green colors were presented from below in an arena, walking flies learned both the appetitive (based on pairing one color with sugar) and the aversive discrimination (based on pairing one color with electric shock) but failed if MB function was blocked using neurogenetic tools (Vogt et al., 2014). Furthermore, MBs are required for visual context generalization (e.g., generalizing landmark discrimination in a flight simulator in which contextual light was switched from blue to green between training and test) (Liu et al., 1999; Tang and Guo, 2001; Brembs and Wiener, 2006). Thus, MBs participate in different forms of visual learning in fruit flies, although their involvement in these phenomena seems to be less clear than in other insects.

Taken together, our results revealed that learning a visual discrimination under a 2D VR, in which closed-loop conditions restricted stimulus control to lateral displacements, induced a neural signature that spanned the optic lobes and MB calyces and that was characterized by IEG downregulation, consistent with an inhibitory trace. This trace may vary and become excitatory in more permissive VR conditions in which closed-loop conditions allow for 3D exploration during discrimination learning (Geng et al., 2022).

## Materials and Methods

Honey bees (*Apis mellifera*) were obtained from our apiary located at the campus of the University Paul Sabatier – Toulouse III during September 2021. Only foragers caught upon landing on a gravity feeder filled with a 0.9 M sucrose solution were used in our experiments to ensure high appetitive motivation. Captured bees were enclosed in individual glass vials and then transferred to small cages housing ten bees in average; caged bees had access to *ad libitum* water and to 300 μl of 1.5 M sucrose solution. They were kept overnight in an incubator at 28°C and 80% humidity. On the next day, they were placed on ice for five minutes to anesthetize them and facilitate the gluing of a tether to their thorax by means of melted wax (Figure 1A). After being attached to the tether, each bee was placed on a small (5 cm diameter) Styrofoam ball for familiarization with the treadmill situation. Bees were provided with 5 μl of 1.5 M sucrose solution and kept for 3 h in this provisory setup in the dark. They were then moved to the VR arena and used for the experiments.

Once in the VR setup, the bee was attached to a holder that allowed adjusting its position on the treadmill (Figure 1B), a polystyrene ball (diameter: 5 cm, weight: 1.07 g) held by a 3D-printed support and floating on a constant airflow produced by an air pump (airflow: 555 ml/s; Aqua Oxy CWS 2000, Oase, Wasquehal, France).

### Virtual Reality Setup

The VR setup consisted of the treadmill and of a half-cylindrical vertical screen made of semi-transparent tracing paper, which allowed presentation of a 180° visual environment to the bee (diameter: 268 mm, height: 200 mm, distance to the bee: 9 cm Figures 1A–C) and which was placed in front of the treadmill. The visual environment was projected from behind the screen using a video projector connected to a laptop (Figure 1A). The video projector was an Acer K135 (Lamp: LED, Maximum Vertical Sync: 120 Hz, Definition: 1280 × 800, Minimum Vertical Sync: 50 Hz, Brightness: 600 lumens, Maximum Horizontal Sync: 100.10^3^ Hz, Contrast ratio: 10 000:1, Minimum Horizontal Sync: 30.10^3^ Hz) (Buatois et al., 2018). The movements of the walking bee on the treadmill were recorded by two infrared optic-mouse sensors (Logitech M500, 1000 dpi, Logitech, Lausanne, Switzerland) placed on the ball support perpendicular to each other.

Experiments were conducted under 2D closed-loop conditions, i.e., rotations of the ball displaced the visual stimuli only laterally. To this end, we used a custom software developed using the Unity engine (version 2018.3.11f1), open-source code available at https://github.com/G-Lafon/BeeVR (Lafon et al., 2021). The software updated the position of the bee within the VR every 0.017 s.

### Visual Stimuli

Bees had to discriminate two vertical rectangles (Figure 1C) based on their different colors and association with reward and punishment. The colors of the rectangles (see Supplementary Figure 1) were blue (RGB: 0, 0, 255, with a dominant wavelength of 446 nm and an irradiance of 161000 μW) and green (RGB: 0, 100, 0, with a dominant wavelength of 528 nm and an irradiance of 24370 μW/cm^2^). They were displayed on a black background (RGB: 0, 0, 0). These colors were chosen based on previous work showing their successful learning in the VR setup (Buatois et al., 2018; Lafon et al., 2021).

Each rectangle had a 5 cm base and occupied the entire vertical extent of the screen. The rectangles were positioned at −50° and + 50° from the bee’s body axis at the beginning of each trial (Figure 1D, left). Keeping the object within −12.5° and + 12.5° in front of the central axis of the bee continuously for 1 s was recorded as a choice (Figure 1D, right).

### Conditioning and Testing at the Treadmill

Bees were trained using a differential conditioning, which promotes better learning performances owing to the presence of penalized incorrect color choice that result in an enhancement of visual attention (Avarguès-Weber and Giurfa, 2014).

Bees were trained during 10 consecutive trials using a differential conditioning procedure (Figure 1E) in which one of the rectangles (i.e., one of the two colors, green or blue) was rewarded with 1.5 M sucrose solution (the appetitive conditioned stimulus or CS +) while the other rectangle displaying the alternative color (the aversive conditioned stimulus or CS−) was associated with 3 M NaCl solution. The latter was used to increase the penalty of incorrect choices (Ayestaran et al., 2010; de Brito Sanchez et al., 2015; Aguiar et al., 2018; Bestea et al., 2021). To avoid directional biases, the rewarded and the punished color rectangles were swapped between the left and the right side of the virtual arena in a pseudo random manner along trials.

At the beginning of the experiment, bees were presented with a dark screen. During training trials, each bee faced the two rectangles (Figure 1D, left). Choice of the CS + rectangle was recorded if the bee kept it at the center of the screen (between −12.5° and + 12.5° of the bee’s central axis) during 1 s (Figure 1D, right). Training was balanced in terms of color contingencies (i.e., blue and green equally rewarded across bees) based on a random assignment by the VR software. If the bee kept the CS + in the center of the screen continuously during 1 s (i.e., if it chose it), the screen was locked on that image for 8 s. This allowed the delivery of sucrose solution in case of a correct choice, or of NaCl in case of an incorrect choice. Solutions were delivered for 3 s by the experimenter who sat behind the bee and used a toothpick to this end. The toothpick contacted first the antennae and then the mouthparts while the screen was locked on the visual image fixated by the bee. A different toothpick was used for each tastant. Each training trial lasted until the bee chose one of the two stimuli or until a maximum of 60 s (no choice). Trials were separated by an inter-trial interval of 60 s during which the dark screen was presented. Bees that were unable to choose a stimulus (i.e., that did not fulfill the criterion of a choice defined above) in at least 5 trials were excluded from the analysis. From 50 bees trained, 40 were kept for analysis (∼80%).

After the last training trial, each bee was subjected to a non-reinforced test that lasted 60 s (Figure 1E). Test performance allowed distinguishing *learners* (i.e., bees that chose the CS + as their first choice in the test) from *non-learners* (i.e., bees that either chose the CS− in their first test choice or that did not make any choice during the test). IEG expression was compared between these two groups, which had the same sensory experience in the VR setup and which differed only in their learning success.

### Brain Dissection

One hour after the test, the bee was sacrificed and its head was instantly frozen in a nitrogen solution. The frozen head was dissected on dry ice under a binocular microscope. First, the antennae were removed and a window was cut in the upper part of the head capsule, removing the cuticle between the compound eyes and the ocelli. Second, the glands and tracheae around the brain were removed. Third, the retinas of the compound eyes were also removed.

The frozen brain was cut in three main parts for IEG analyses (Figure 3A): the optic lobes (OL), the upper part of the mushroom bodies (the mushroom-body calyces, MB Ca) and the remaining central brain (CB), which included mainly the peduncula of the mushroom-bodies (a and b lobes), the central complex (CC), the antennal lobes (AL) and the subesophageal zone (SEZ). Samples were stored at −80°C before RNA extraction. During the dissection process, one *learner* brain was lost so that learner sample sizes differ between the behavioral (*n* = 23) and the molecular analyses (*n* = 22).

### RNA Extraction and Reverse Transcription

The RNAs from the three sections mentioned above (OL, MB Ca and CB) were extracted using the RNeasy Micro Kit (Qiagen). The final RNA concentration obtained was measured by spectrophotometry (NanoDrop One, Thermo Fisher Scientific). A volume of 10 μl containing 100 ng of the RNA obtained was used for reverse transcription following the procedure recommended in the Maxima H Minus First Strand cDNA Synthesis kit (Thermo Fisher Scientific, 0.25 μl of random hexamer primer, 1 μl of 10 mM dNTP mix, 3.75 μl of nuclease free H_2_O, 4 μl 5× RT Buffer and 1 μl Maxima H Minus Enzyme Mix).

### Quantitative Polymerase Chain Reaction

All the primers used for target and reference genes generated amplification products of approximately 150 bp. The efficiencies of all reactions with the different primers used were between 95 and 110% (Table 1). Their specificity was verified by analyzing melting curves of the RT-qPCR products (see Supplementary Figure 2). Two reference genes (*Ef1a* and *Actin*) were used for normalization.

Expression was quantified using a SYBR Green real-time PCR method. Real-time PCR were carried out in 384-Well PCR Plates (Bio-Rad) cover with Microseal ‘B’ PCR Plate Sealing Film (Bio-Rad). The PCR reactions were performed using the SsoAdvancedTM Universal SYBR Green Supermix (Bio-Rad) in a final volume of 10 μl containing 5 μl of 2× SsoAdvancedTM Universal SYBR Green Supermix, 2 μl of cDNA template (1:3 dilution from the reverse transcription reaction), 0.5 μl of 10 μmol of each primer and 2 μl of ultrapure water. The reaction conditions were as follows: 95°C for 30 s followed by 40 cycles of 95°C for 10 s, 55°C for 30 s and a final step at 95°C for 10 s followed by a melt curve from 55°C to 95°C with 0.5°C per second. The reaction was performed in a CFX384 Touch Real-Time PCR Detection System (Bio-Rad) and analyzed with the software Bio-Rad CFX Manager.

Each sample was run in triplicates. If the triplicates showed too much variability (SD > 0.3), the furthest triplicate was discarded. If the two remaining triplicates still showed too much variability (SD > 0.3) the sample was discarded. The samples were subjected to a relative quantification and normalization. First for each sample and for each reference gene per brain region, the relative quantity (Qr) was computed using the difference between the mean Ct value of each sample and the highest mean Ct value (ΔCt), using the following formula: Qr = (1 + E)^Δ^ *^Ct^* (with E = efficiency of the reaction). Then a normalization factor for each sample was obtained computing the geometric mean of the relative quantities obtained for the reference genes in the corresponding samples (ΔΔCt).

### Data Analysis and Statistics

#### Behavioral Data

The first choice of the bees was recorded during the conditioning trials and the non-reinforced test. In this way, we established for each trial and test the percentages of bees choosing first each of the stimuli displayed or not choosing a stimulus (± 95% confidence interval).

Test percentages were analyzed within groups by means of a generalized linear mixed model (GLMM) for binomial family in which the individual identity (Bee) was considered as a random factor (individual effect) while the choice category (CS +, CS−, and NC) was fitted as a fixed effect; *z* values with corresponding degrees of freedom are reported throughout for this kind of analysis.

For the acquisition trials, we recorded motor variables such as the total distance walked during a trial, and the walking speed. The analysis of these continuous variables was done using a linear mixed model (lmer function) in which the individual identity (*Bee ID*) was a random factor and the factors *Group* (i.e., learner or non-learner) and *Trial* were fixed.

Statistical analyses were performed using with R 3.5.1 (R Development Core Team, 2016). The package lme4 was used for GLMMs and LMMs.

#### Gene Expression Data

Statistical differences in gene expression were assessed for reference genes to check for stability and for target genes within a given brain region using One-Factor ANOVA for independent groups in the case of multiple comparisons or two-sample *t* test in the case of dual comparisons. *Post hoc* comparisons between groups were performed by means of a Tukey test following ANOVA. No cross-comparisons between brain regions or genes were performed due to within-area normalization procedures. Statistical analyses were done either with R 3.5.1 software (R Development Core Team, 2016) or with Statistica 13 Software (TIBCO Data Science).

## Data Availability Statement

The datasets presented in this study can be found in online repositories. The names of the repository/repositories and accession number(s) can be found below: 10.6084/m9.figshare.17705765.v1.

## Author Contributions

AB, AA-W, IM, and MG conceived the project. GL performed all the behavioral experiments and performed statistical analyses on behavioral data. HG dissected and sectioned the brains of the bees trained in the VR setup and performed all the molecular analyses. AA-W and MG supervised behavioral experiments. IM and MG supervised molecular experiments. HG and MG performed statistical analyses on gene-expression data. MG wrote the manuscript and obtained the funding necessary for this work. All authors corrected, discussed, reviewed, and approved the final version of the manuscript.

## Conflict of Interest

The authors declare that the research was conducted in the absence of any commercial or financial relationships that could be construed as a potential conflict of interest.

## Publisher’s Note

All claims expressed in this article are solely those of the authors and do not necessarily represent those of their affiliated organizations, or those of the publisher, the editors and the reviewers. Any product that may be evaluated in this article, or claim that may be made by its manufacturer, is not guaranteed or endorsed by the publisher.
