# Supplementary material for: The Neural Signature of Visual Learning Under Restrictive Virtual-Reality Conditions
Source: Front Behav Neurosci. 2022 Feb 16;16:846076. doi: 10.3389/fnbeh.2022.846076 (PMC8888666; doi:10.3389/fnbeh.2022.846076)
Supplement: Supplementary file 1 [file Image_1.pdf]

Supplementary Materials

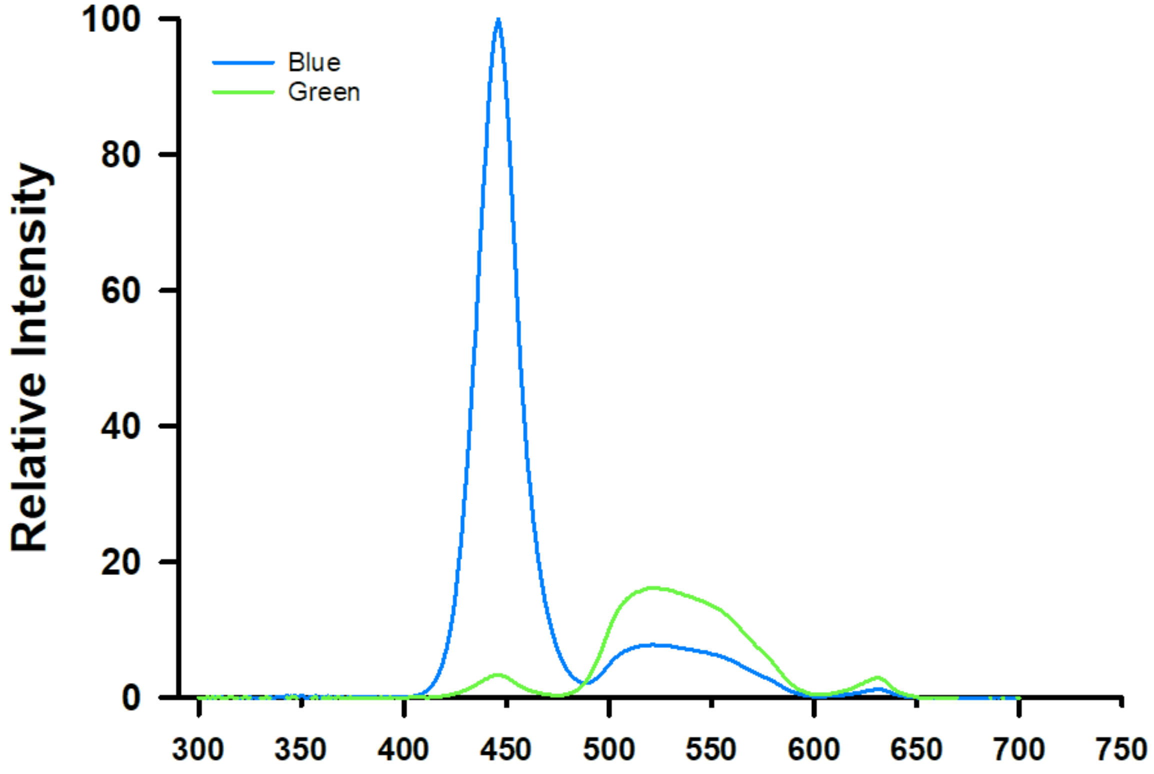

Supplementary Figure 1. Spectral distribution (relative intensity as a function of wavelength) of the blue light (dominant wavelength 446 nm) and the green light (dominant wavelength 528 nm) used to train the bees in the color discrimination task.

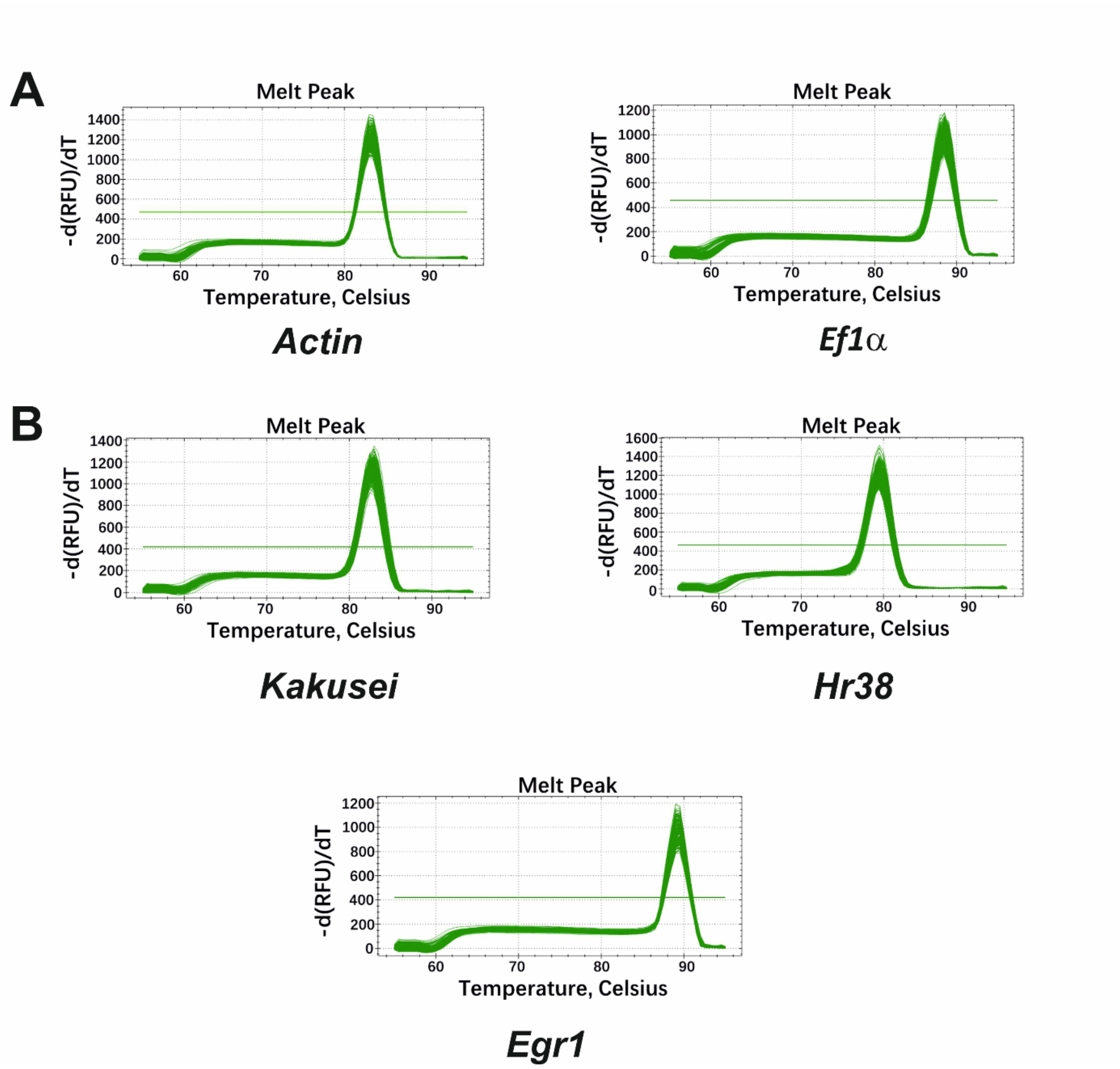

Supplementary Figure 2. Validation selectivity of gene-specific primers. Melting peaks of RT-qPCR. **A)** Reference genes. **B)** Target genes.

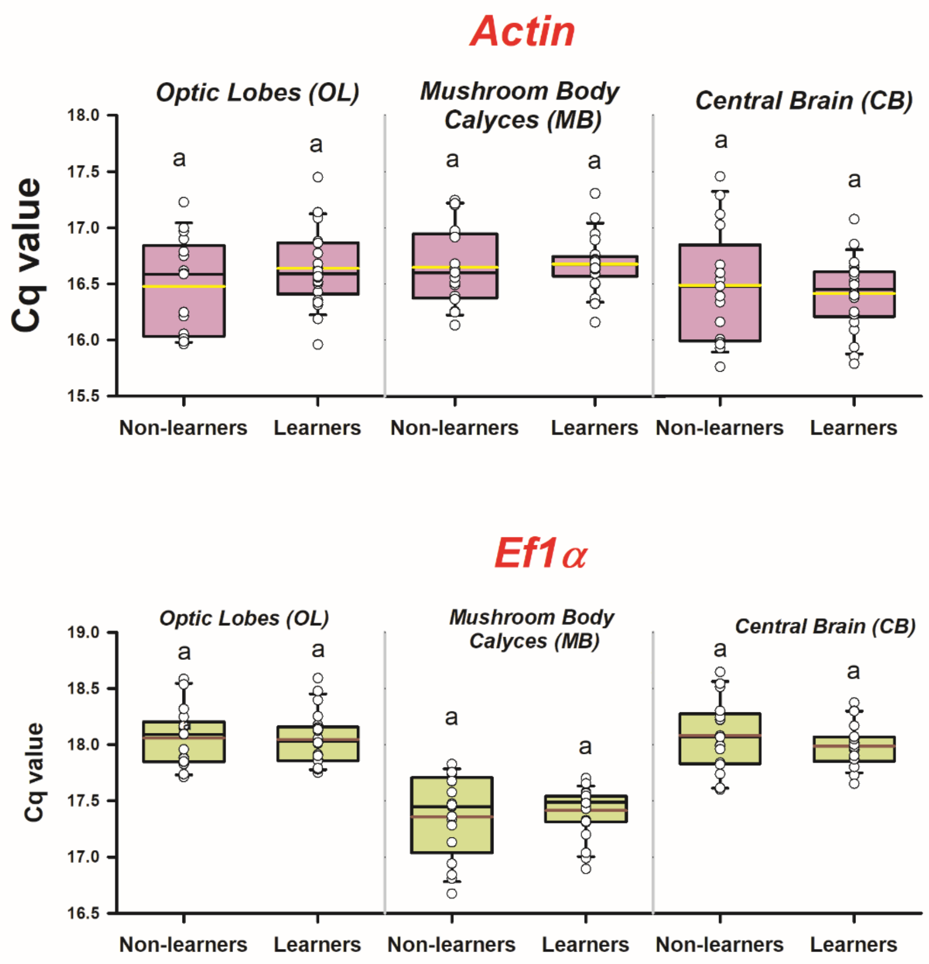

Supplementary Figure 3. Expression levels (Cq values) of the reference genes *Actin* (upper row) and *Ef1α* (lower row). Expression levels in the brain regions considered (optic lobes, mushroom body calyces and central brain) of learners and non-learners (n=22 and n=17, respectively, for both genes). Box plots show the mean value in yellow (*Actin*) or red (*Ef1α*). Sample sizes are indicated within parentheses below each group. Error bars define the 10th and 90th percentiles. Same letters on top of box plots indicate absence of significant differences (two-sample t test; $p < 0.05$).
